# Ostéonécrose aseptique de la tête fémorale chez une patiente atteinte d'artérite de Takayasu

**DOI:** 10.11604/pamj.2014.18.203.4679

**Published:** 2014-07-06

**Authors:** Hanen Loukil, Faten Frikha, Mouna Snoussi, Raida Ben Salah, Zouhir Bahloul

**Affiliations:** 1 Service de Médecine Interne CHU Hédi Chaker, Sfax, Tunisie

**Keywords:** Ostéonécrose aseptique, tête fémorale, artérite de Takayasu, aseptic osteonecrosis, femoral head, Takayasu arteritis

## Abstract

L'ONA n'est pas une maladie spécifique, mais c'est l'aboutissement de diverses conditions pathologiques dont la plupart altère la circulation sanguine. Elle peut compliquer toutes les maladies systémiques et auto-immunes essentiellement le lupus érythémateux systémique. Sa survenue au cours de l'artérite de Takayasu a été exceptionnellement rapportée. Nous rapportons l'observation d'une femme âgée de 69 ans, et suivie pour artérite de Takayasu qui se présentait pour des douleurs de la hanche gauche. Un scanner de la hanche gauche avait confirmé le diagnostic d'une ostéonecrose aseptique de la tête fémorale gauche stade 3.

## Introduction

L'Ostéonecrose aseptique (ONA) est une maladie dont la pathogénie est multifactorielle avec une participation mécanique, métabolique, vasculaire et iatrogène cortisonique [1, 2]. La fréquence de l'association avec des maladies auto-immunes est variable. Son association avec l'artérite de Takayasu (AT) a été exceptionnellement rapportée [3]. Nous rapportons une nouvelle observation d'ONA chez une patiente suivie pour une maladie de Takayasu. Nous essayerons à travers cette observation et une revue de la littérature d’étayer les mécanismes physiopathologiques, les moyens diagnostiques et la prise en charge thérapeutique de l'ONA au cours de cette vascularite.

## Patient et observation

Une femme âgée de 69 ans, hypertendue, dyslipidémique, et coronarienne, suivie pour AT (phénomène de Raynaud, Hypertension artérielle avec asymétrie tensionnelle, syndrome inflammatoire biologique et aspect échographique et angiographique typique d'artérite de Takayasu par atteinte des troncs supra-aortiques) depuis 1985 traitée par corticoïdes, se présentait pour des douleurs sévères touchant la hanche gauche. L'examen physique objectivait une douleur sévère à la mobilisation et une limitation de l'abduction et de la flexion de la hanche.

**Figure 1:**
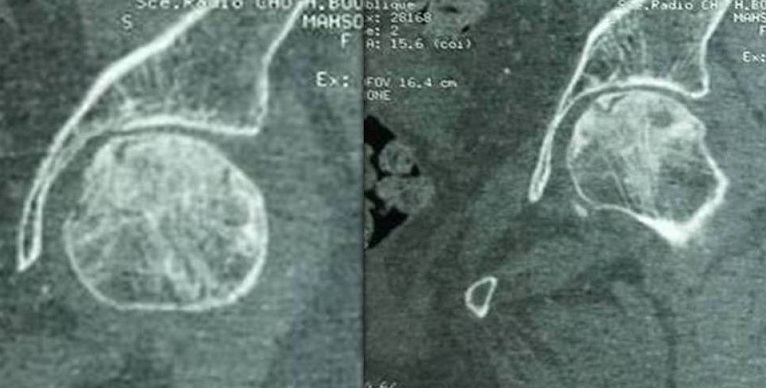
Tomodensitométrie de la hanche gauche: lésions géodiques épiphysaires avec un liseré clair de dissection sous chondrale évocateur d'une ostéonecrose aseptique de la tête fémorale gauche stade 3

La radiographie du bassin objectivait un aspect en coquille d'oeuf de la tête fémorale droite et un pincement de l'articulation coxo-fémorale gauche sans images géodiques. La scintigraphie osseuse montrait une hyperfixation de la tête fémorale gauche et du condyle fémoral externe droit. L'Imagerie par résonance magnétique n’était pas réalisée (patiente porteuse de stent). Un scanner de la hanche gauche avait montré des lésions géodiques épiphysaires avec un liseré clair de dissection sous chondrale allant d'une corticale à une autre évocateur d'une ostéonecrose aseptique de la tête fémorale gauche stade 3 (Figure 1). La patiente était traitée par décharge, un traitement antalgique simple avec diminution des douleurs et persistance d'une gêne fonctionnelle dans la vie quotidienne.

## Discussion

L'ONA n'est pas une maladie spécifique, mais c'est l'aboutissement de diverses conditions pathologiques dont la plupart altère la circulation sanguine [1]. Elle peut être idiopathique dans 40% des cas ou secondaire à plusieurs étiologies: post-traumatique, la corticothérapie, l'alcoolisme, la maladie de Gaucher, les barotraumatismes, la drépanocytose, les maladies systémiques, etc..[1–3].

L'ONA peut compliquer toutes les maladies systémiques et auto-immunes essentiellement le LES [3, 4]. Sa fréquence de survenue au cours de ces maladies est variable. L'ONA représente une complication redoutable, dont les mécanismes physiopathologiques sont complexes dominés par la corticothérapie prolongée [3, 5]. Néanmoins, une ostéonécrose peut survenir au cours d'une maladie systémique en dehors de toute corticothérapie. L'hypothèse d'une vascularite des vaisseaux épiphysaires et/ou la présence d'anticorps antiphospholipides a été invoquée.

L'AT est une vascularite inflammatoire, sténosante des artères de moyen et de gros calibre, atteignant électivement la femme jeune. L'occlusion des petits vaisseaux est une complication microcirculatoire rare de la maladie de Takayasu pouvant être responsable d'ischémie et de nécrose osseuse. La survenue d'une ONA au cours de cette vascularite a été exceptionnellement rapportée [3, 5]. Dans l’étude de Shigemura et al. [5] incluant 302 patients avec différentes maladies auto-immunes et ayant tous reçu une corticothérapie, une ONA était observée dans 37% parmi 173 cas de LES; dans 13 cas parmi 27 cas de PM/DM; dans 3 cas parmi 14 cas de vascularites type AT, dans 3 cas parmi 5 cas de maladie de Still de l'adulte, dans 1 cas parmi 3 cas de Sclérodermie systémique et dans 1 cas parmi 3 cas de granulomatose avec polyangéite (anciennement appelée Granulomatose de Wegener). Aucun cas d'ONA n'a été observé des 5 cas de polyarthrite rhumatoïde, des 3 cas de sarcoïdose et des 2 cas de maladie de Behçet.

Quelque soit l’étiologie de l'ONA, les signes cliniques sont inconstants, aspécifiques et seraient fonction du stade de la nécrose. L´apparition des manifestations cliniques peut être précédée d´une phase de nécrose latente d´une durée variable [1, 2]. Les signes d'appel de l'ONA sont variables selon les études, mais la douleur constitue le maitre symptôme. Le début peut être marqué par une douleur brutale qui s´atténue par la suite. Le plus souvent, elle est d´installation progressive, initialement mécanique pouvant devenir ultérieurement permanente.

Les facteurs qui pourraient induire une ONA chez ces patients atteints d'une vascularite incluent le phénomène de Raynaud, la vascularite, le syndrome des antiphospholipides, les emboles graisseux et le traitement par corticoïdes. En fait, La corticothérapie est une cause majeure de l'ONA [1, 4]. La responsabilité de la corticothérapie dans la survenue des nécroses osseuses est bien établie depuis 1957. Il est estimé que 30% des ONA surviennent chez des patients recevant une corticothérapie prolongée. Les glucocorticoïdes stimulent et régulent l'adipogenèse dans les cellules stromales de la moelle osseuse. La réponse adipogénique aux stéroïdes correspond à une hypertrophie et à une hyperplasie des cellules graisseuses [6]. Cette hypertrophie et hyperplasie des adipocytes de la moelle osseuse va entrainer une augmentation de la pression intra osseuse et une baisse du flux sanguin pouvant jouer un rôle important dans l'ON induite par les stéroïdes. L'embolie ou la thrombose induite aussi par les stéroïdes par le biais de perturbation de la coagulabilité, favorise davantage la formation de microembolies de lipides et par suite ce type d'ischémie [7]. L'ONA de la tête fémorale est le siège préférentiel des nécroses cortisoniques. Dans les différentes cohortes, sa fréquence est souvent supérieure à 50%. Néanmoins, notre patiente avait une longue durée de maladie (15 ans), traitée par corticoïdes associées à d'autres facteurs de risques: dyslipidémie. Il semble que la cause de l'ONA chez notre patiente soit multifactorielle mais favorisée essentiellement par la prise prolongée de corticoïdes. De ce fait, l'utilisation rationnelle des corticoïdes est obligatoire et un diagnostic précoce de cette complication pourrait améliorer le pronostic.

Parmi les 4 examens complémentaires dans l'exploration de l'ONA à savoir la radiologie standard, la scintigraphie, le scanner et l'IRM, la radiologie standard est toujours en retard par rapport aux autres méthodes d'imageries [1]. C'est ainsi que plusieurs auteurs classent ces différentes techniques dans le diagnostic précoce de l'ONA par ordre de performance en IRM, Scanner puis scintigraphie osseuse et enfin radiographie standard. A l'heure actuelle, l'IRM est la technique la plus précoce et la plus sensible dans le dépistage de l´ostéonécrose. Le signe le plus constamment décrit est un liseré de bas signal limitant complètement la zone de nécrose et dont les extrémités atteignent la lame osseuse sous-chondrale. Le liseré matérialise l'interface entre le séquestre et l'os sain. Chez notre patiente, la réalisation de l'IRM était contre indiquée par le port de Stent et nous avons réalisé une TDM qui avait confirmé le diagnostic de l'ONA. Le scanner n´est pas très performant pour la détection précoce de l'ON puisqu´il montre exclusivement les modifications de la trame osseuse qui apparaissent tardivement comme chez notre patiente qui avait une ONA stade 3.

Le traitement de l'ostéonécrose des patients ayant une maladie systémique est identique à celui des ostéonécroses « classiques ». Le but de ce traitement est de préserver l'intégrité articulaire. Le choix thérapeutique au cours de l'ONA dépend du stade de la maladie, de l’étendue de la nécrose, également du siège et de l’âge du patient et de son état général. Le meilleur facteur pronostic du traitement reste sans doute la précocité du diagnostic.

## Conclusion

Malgré des mécanismes physiopathologiques distincts, l'association entre la maladie de Takayasu et l'ONA ne semble pas fortuite et pourrait être sous-tendue par une complication microcirculatoire rare de la maladie de Takayasu ou iatrogène secondaire à la corticothérapie prolongée.
